# Evaluating lymphocyte change rate and lactate as predictors of prognosis in critical COVID-19 patients in the intensive care unit

**DOI:** 10.3389/fcimb.2025.1532174

**Published:** 2025-10-16

**Authors:** Yuxiu Tang, Jin Yang, Liquan Chen, Xueke Liu, Zhen Chen, Jiaxi Lin, Jun Jin, Yao Wei

**Affiliations:** ^1^ Department of Intensive Care Unit, The Second Affiliated Hospital, Hengyang Medical School, University of South China, Hengyang, China; ^2^ Department of Intensive Care Unit, The First Affiliated Hospital of Soochow University, Suzhou, China

**Keywords:** coronavirus disease 2019, lactate, lymphocyte change rate, outcome, intensive care unit

## Abstract

**Background:**

Studies have shown that lymphocyte counts and lactate (LAC) levels have a certain relationship with the prognosis of COVID-19 patients. In the present study, we aimed to determine the predictive effects of new indicator lymphocyte change rate and LAC on the prognosis of COVID-19 patients.

**Methods:**

In the present study, we retrospectively analyzed the clinical data of 137 adult patients (≥ 18 years old) diagnosed with the COVID-19 Omicron variant, who were admitted to the comprehensive, respiratory, or infection ICU of our hospital, between November 2022 and February 2023. Cox regression and causal mediation analyses were used to evaluate the relationship between the clinical test data and patient prognosis.

**Results:**

A total of 137 patients with COVID-19 were included in the present study, 77.40% of whom were male, with an average age of 73 years and an overall mortality rate of 51.8%. Multifactorial logistic regression analysis showed that LAC (odds ratio [OR], 0.05; 95% confidence interval [CI], 0–0.1; *P* = 0.047] and the weekly rate of change in LYM (change in LMY over the course of a week/LYM on the day of admission) had a good predictive value for the prognosis of patients, while respiratory-related indicators had no significant relationship with prognosis (P > 0.05). The combined predictive value of LAC and the weekly rate of change in LYM was even greater, with an area under the curve of 0.7629. In terms of prognosis, 1.75 mmol/L was set as the cut-off value for LAC (sensitivity, 57.7%; specificity, 75.8%). The mortality rate of patients with LAC > 1.75 mmol/L was significantly higher than that those with LAC < 1.75 mmol/L, and NLR was found to have a mediating effect in lactate-mediated death.

**Conclusion:**

In summary, lymphocyte change rate combined with LAC had the greatest predictive value for COVID-19 ICU patient prognosis, while respiratory-related indicators had no significant relationship with prognosis, so we suggest the increase of LAC in COVID-19 patients may be caused by microcirculatory disturbances.

## 1 Introduction

Coronavirus disease 2019 (COVID-19) was first encountered in Wuhan, China in December 2019, as a case of unexplained pneumonia caused by the novel severe acute respiratory syndrome coronavirus 2 (SARS-CoV-2), which was quickly followed by a global outbreak that was declared a pandemic by the World Health Organization (WHO) on March 11, 2020 (Guan et al., 2020; Zhu et al., 2020). According to the WHO, as of the time of the writing of this article, there have been 766,440,796 confirmed cases of COVID-19 worldwide, resulting in 6,932,591 deaths (WHO COVID-19 Dashboard, 2020). COVID-19, therefore, poses a serious threat to public health worldwide[3]. Viruses are highly diverse, due to their susceptibility to mutations and recombination (Huo et al., 2020), and during the last three years of the COVID-19 pandemic, numerous variants have been identified by the WHO, including Alpha, Beta, Gamma, Delta, and Omicron. The common mechanism by which COVID-19 invades the body is by targeting angiotensin-converting enzyme 2 (ACE2), exerting a pathogenic effect (Mathioudakis et al., 2022).

Lactate (LAC) is the final product of cellular glycolysis under anaerobic conditions, and is a metabolic waste product removed from the blood by the liver and kidneys (Rabinowitz and Enerbäck, 2020). Conventional wisdom has been that increased LAC levels are due to anaerobic metabolism caused by hypoxia in the body. Bakker et al (Bakker et al., 2013; Gupta, 2022) proposed that the cause of hyperlactatemia may be multifactorial, as it can also occur under completely aerobic conditions. It has been shown that the level of LAC can be significantly higher in patients hospitalized with COVID-19 compared to those that are not hospitalized, that the initial LAC level in deceased patients is higher than that in those that survive (Trebuian et al., 2024; Velavan et al., 2021), and that lactate dehydrogenase (LDH), a key enzyme in the conversion of pyruvate to LAC, can be used as a significant independent risk factor for predicting the occurrence respiratory failure in severe COVID-19 patients (Wang et al., 2024). Based on a multifactorial analysis of the increase in LAC levels, it is necessary to further explore whether the increase in LAC in patients with COVID-19 is due only to hypoxia from impaired ventilation and air exchange resulting from pulmonary changes, or if it is also due to a combination of local ischemia, which may result in an imbalance in the ventilation-to-blood flow ratio.

Significant changes can be observed in the immune systems of patients with COVID-19, which primarily manifest as a continuous and significant decrease in peripheral blood lymphocyte (LYM) count, the majority of which are CD4+T and CD8+T cells. These changes are significantly related to complications and poor prognoses (Zhou et al., 2020); therefore, changes in the LYM count should be dynamically observed in clinical practice. As such, we proposed using the rate of change in LYM over the course of one week (changes in LYM counts during the first week after admission)/(LYM counts on the day of admission) to determine the predictive value of LYM count for prognoses. LYM counts and its correlation with other test indicators, such as Neutrophil (NEU)-to- Lymphocyte ratio (NLR), Platelet-to- Lymphocyte ratio (PLR), and Lymphocyte-to-Monocyte ratio (LMR) have been studied extensively, of which NLR is considered an independent predictor of the risk of death in COVID-19 patients (Colaneri et al., 2024), with a higher ratio being associated with an increased risk of thrombosis (Pivina et al., 2024).

The present study, therefore, aimed to explore the predictive value of LAC and the weekly rate of change in LYM on the prognosis of patients, through a retrospective analysis of the clinical data of COVID-19 patients admitted to the intensive care unit (ICU), and whether there was any relationship with other clinical indicators.

## 2 Materials and methods

### 2.1 Study design

The following data were collected from 137 comprehensive, respiratory, and infection ICU patients with reverse transcription polymerase chain reaction (RT-PCR) assay-confirmed COVID-19: clinical demographic, vital signs (first and seventh day of ICU admission), related laboratory test results, clinical treatment and outcomes, and Acute Physiology and Chronic Health Evaluation II (APACHE II) and Sequential Organ Failure Assessment (SOFA) scores (first and seventh days of ICU admission). All data were obtained from the hospital’s electronic medical record database during the initial implementation of the re-opening policy in China from November 2022 to February 2023.

### 2.2 Statistical analysis

The basic data from 168 ICU patients were processed, and after excluding patients with incomplete data, a total of 137 patients were included for analysis in the present study. The total number and proportions were used to represent categorical variables. For normally distributed variables, the data were expressed as mean and standard deviation (SD), while skewed data were expressed as median and interquartile range (Q1–Q3). The Chi-squared or Fisher’s exact test was used to compare the categorical variables, the student’s t-test was used to compare the normally distributed continuous variables, the Mann-Whitney U test was used to compare the skewed continuous variables, and multiple regression was selected to characterize the relationship between clinical indicators and primary outcomes. Baseline variables which were clinically relevant or had a univariate relationship with prognosis, such as age, fraction of inspired oxygen (FiO_2_), LAC level, white blood cell (WBC) count, NEU count, NLR, LDH, D-dimer (DD) level, fibrinogen degradation product (FDP), and APACHE II and SOFA scores, were entered into the multivariate logistic regression model as covariates. The receiver operating characteristic (ROC) curve and the area under the curve (AUC) were calculated to evaluate and compare the diagnostic performance of each parameter. A restricted cubic spline (RCS) was used to predict the relationship between LAC level and event risk, and the inflection point was set as the cut-off point. Stratified based on the LAC cut-off value in the ROC curve, the relationship of LAC level with associated factors, including APACHE II and SOFA scores on the seventh day as well as hospitalization time, was evaluated using the Cox proportional risk model, with covariates adjusted for sex and age for each model. We created a survival curve stratified based on LAC values, after which we conducted a causal mediation analysis (CMA) to determine whether other clinical indicators changed the relationship between LAC level and clinical outcomes. For this, we determined which indicators were simultaneously related to LAC level and clinical outcomes, and the shortlisted indicator, NLR, was subjected to CMA as described by previous studies (Huang and Yang, 2017; Choy et al., 2023), as an instrumental variable, with LAC level as the independent variable and clinical outcomes as the dependent variable. Statistical analyses were performed using R software version 4.2.0. (R Foundation for Statistical Computing) with statistical significance set at *P* < 0.05.

## 3 Results

### 3.1 Basic clinical characteristics of patients

A total of 137 patients were included in the present study, 77.40% of whom were male, with an average age of 72.7 years. The mortality rate was 51.8%, with the majority of deaths being men (84.5%), and the average length of stay in the ICU was 20 days. The age of the deceased patients, as well as their APACHE II and SOFA scores on the day of ICU admission were significantly higher than those of the surviving patients, although their hospitalization time was shorter. FIO_2_, LAC level, WBC count, NEU count, NLR, blood urea nitrogen (BUN), aspartate aminotransferase (AST), alanine aminotransferase (ALT), LDH, FDP, and DD in the deceased patients were significantly higher (*P* < 0.05), while their albumin levels were significantly lower (*P* = 0.047) than those in the surviving patients (**Table 1**). The LYM count in the surviving patients showed a statistically significant upward trend within one week of ICU admission (*P* < 0.001). Multivariate regression analysis showed that age, LAC level, NLR, and SOFA score were significantly associated with the patients’ prognoses (*P* < 0.05) (**Table 2**).

**Table 1:** Basic clinical characteristics of patients.

Variables	Total (n = 137)	Survival (n = 66)	Non-survival (n = 71)	*p*
Demographic and clinical characteristics				
Age (years), Median (Q1,Q3)	76 (66, 82)	72.5 (62.25, 80)	79 (70, 82.5)	0.003^*^
sex, n (%)				0.062
Female, n (%)	31 (23)	20 (30)	11 (15)	
Male, n (%)	106 (77)	46 (70)	60 (85)	
APACHII, Median (Q1,Q3)	15 (10, 19)	11 (8, 16)	17 (12.5, 21)	< 0.001^*^
SOFA, Median (Q1,Q3)	5 (3, 9)	4 (2, 6)	8 (4, 11)	< 0.001^*^
Time, Median (Q1,Q3)	16 (8, 28)	20.5 (12.25, 39.75)	11 (6, 18.5)	< 0.001^*^
Blood gas analysis				
RR, Median (Q1,Q3)	20 (16, 25)	18 (16, 23.75)	20 (17, 25.5)	0.15
FIO2(%), Median (Q1,Q3)	60 (43, 80)	55.5 (41, 70)	60 (50, 80)	0.022^*^
PO2(mmHg), Median (Q1,Q3)	78 (59.5, 125.8)	88.2 (61.65, 150.25)	74.3 (55.4, 110)	0.087
PCO2(mmHg), Median (Q1,Q3)	38.5 (33.5, 46.9)	37.15 (33.2, 42.35)	40 (35.25, 49.25)	0.098
SO2, Median (Q1,Q3)	95.6 (89.8, 98.8)	96.95 (92.03, 99.33)	94.2 (87.85, 97.85)	0.006^*^
LAC(mmol/L), Median (Q1,Q3)	1.5 (1.1, 2.5)	1.25 (1, 1.67)	2.1 (1.3, 3)	< 0.001^*^
<1.75	59 (43)	28 (42)	31 (44)	
>1.75	78 (57)	38 (58)	40 (56)	
PF(mmHg), Median (Q1,Q3)	149 (103, 254)	169 (117.25, 335.75)	131 (82.5, 184.5)	0.004^*^
Laboratory tests				
WBC(×109/L), Median (Q1,Q3)	9.22 (5.93, 12.66)	7.62 (5.4, 11.72)	10.7 (6.8, 14.62)	0.006^*^
NEU(×109/L), Median(Q1,Q3)	8.12 (5.04, 11.79)	6.62 (4.67, 10.25)	9.78 (5.64, 13.44)	0.004^*^
LYM(×109/L), Median (Q1,Q3)	0.53 (0.34, 0.85)	0.5 (0.31, 0.79)	0.56 (0.38, 0.93)	0.141
NLR(×109/L), Median (Q1,Q3)	7.9 (4.7, 11.5)	5.9 (4.05, 9.55)	9.6 (5.12, 13.2)	< 0.001*
LYMrate, Median (Q1,Q3)	0.24 (-0.44, 0.76)	0.46 (0.03, 1.24)	-0.3 (-0.62, 0.38)	< 0.001*
CRP(mg/L), Median (Q1,Q3)	93.5 (36.99, 154.24)	76.41 (28.49, 141.16)	109.74 (57.61, 167.55)	0.058
PLT(×109/L), Median (Q1,Q3)	169 (103, 219)	175 (132, 222.75)	142 (93, 214.5)	0.112
BUN(mmol/L), Median (Q1,Q3)	9.9 (6.98, 17.54)	9.16 (6.02, 12.52)	11.66 (8.09, 19.3)	0.001^*^
Cr(umol/L), Median (Q1,Q3)	76.2 (56.3, 114.3)	71 (54.65, 106.25)	83 (57.1, 126.65)	0.216
ALB(g/L), Median (Q1,Q3)	30.3 (26.9, 33.4)	30.85 (28.33, 34.38)	29.8 (25.65, 32.35)	0.047^*^
ALT(U/L), Median (Q1,Q3)	25 (16.8, 39.5)	22.5 (15, 32.75)	27.5 (19, 47)	0.035^*^
AST(U/L), Median (Q1,Q3)	34.1 (22, 54)	29.2 (20.32, 42.75)	41 (23.35, 68)	0.031^*^
TB(umol/L), Median (Q1,Q3)	12.6 (8.2, 17)	11.45 (8.03, 15.17)	12.9 (8.9, 22.3)	0.126
LDH(U/L), Median (Q1,Q3)	381.7 (232.4, 502.6)	283.8 (202.48, 432.7)	443.7 (320.05, 568.6)	< 0.001^*^
PT (sec), Median (Q1,Q3)	14.4 (13.5, 16.1)	14.2 (13.33, 15.95)	14.5 (13.6, 16.45)	0.272
APTT(sec), Median (Q1,Q3)	37.8 (33, 43.9)	37.35 (33.52, 42.77)	38.1 (32.6, 46.35)	0.613
INR, Median (Q1,Q3)	1.15 (1.07, 1.31)	1.14 (1.06, 1.29)	1.18 (1.08, 1.34)	0.253
FIB(g/L), Mean ± SD	4.55 ± 1.85	4.85 ± 1.69	4.27 ± 1.96	0.065
FDP(mg/L), Median (Q1,Q3)	8.19 (4.13, 21.87)	7.37 (3.24, 14.48)	9.42 (5.36, 31.62)	0.011^*^
DD(ug/mL), Median (Q1,Q3)	2.86 (1.46, 7.3)	2.25 (1.15, 4.58)	4.69 (2.21, 10.26)	0.001^*^

*p-value < 0.05. Data are expressed as number of patients (n), percentages of total related variable (%) and mean ± SD for normally distributed variables and median (IQR) for skewed data. Patients were divided into 2 groups, depending on 28-day ICU mortality. APACHE, acute physiology and chronic health evaluation; SOFA, sequential organ failure assessment; RR, respiratory rate; FiO_2_, fraction of inspired oxygen; PO_2_, arterial partial pressure of oxygen; PCO_2,_ partial pressure of carbon dioxide; PF, PO2/FiO2; WBC, white blood cell; NEU, neutrophils; LYM, lymphocyte; NLR, neutrophil to lymphocyte ratio; LYM rate, the rate of change in lymphocyte within one week (Changes in lymphocyte during the week/lymphocyte counts on the first day);CRP, C-reactive protein; PLT, Platelet count; BUN, blood urea nitrogen; Cr, Creatinine; ALB, albumin; ALT, alanine aminotransferase; AST, aspartate aminotransferase; TB, total bilirubin; LDH, lactate dehydrogenase; PT, prothrombin time; APTT, activated partial thromboplastin time; INR, international normalized ratio; FDP, fibrin degradation product; FIB, fibrinogen; DD, D-Dimer.

**Table 2:** Results of multivariate regression analysis.

Variable	OR(95%CI)	*P*
Age	0.01(0~0.02)	0.003*
FIO2	0(-0.01~0)	0.093
SO2	-0.01(-0.02~0)	0.256
LAC	0.05(0~0.1)	0.047*
PF	0(0~0)	0.535
WBC	0(-0.06~0.05)	0.909
NEU	-0.02(-0.1~0.06)	0.638
NLR	0.03(0~0.06)	0.046*
LYMrate	-0.05(-0.01~0)	0.05
BUN	0(0~0.01)	0.29
ALB	0(-0.01~0.01)	0.88
ALT	0(0~0)	0.222
AST	0(0~0)	0.097
LDH	0(0~0)	0.265
DD	0(-0.01~0.02)	0.422
FDP	0(0~0)	0.522
APACHII	0.02(0~0.03)	0.05
SOFA	0.02(0~0.04)	0.025*

*p-value < 0.05.

### 3.2 Relationship between LAC and prognosis

**Figure 1:**
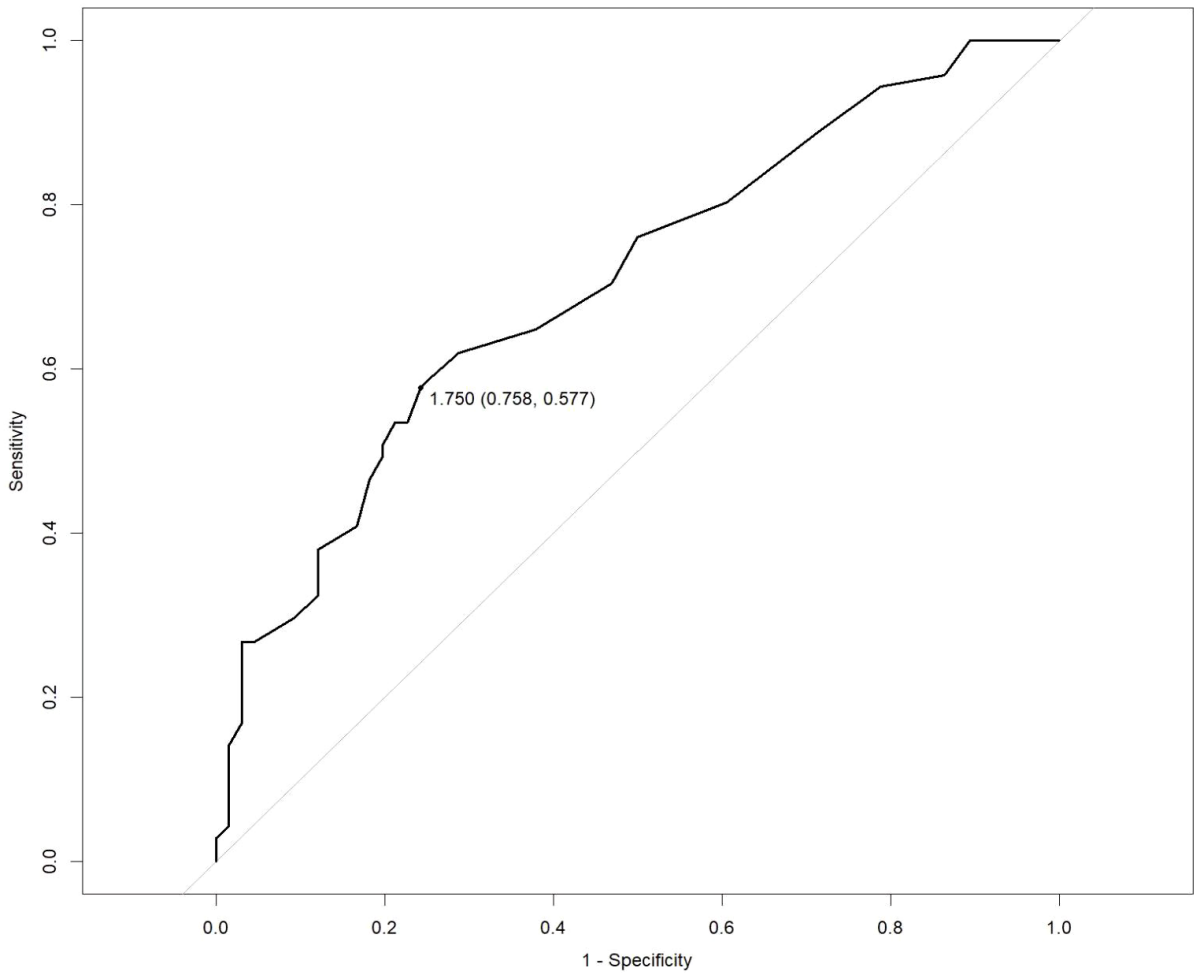
Receiver operating characteristic (ROC) curve for predicting the prognosis of severe COVID-19. LAC: cut-off 1.75 mmol/L, sensitivity 57.7%, specificity 75.8%.

**Figure 2:**
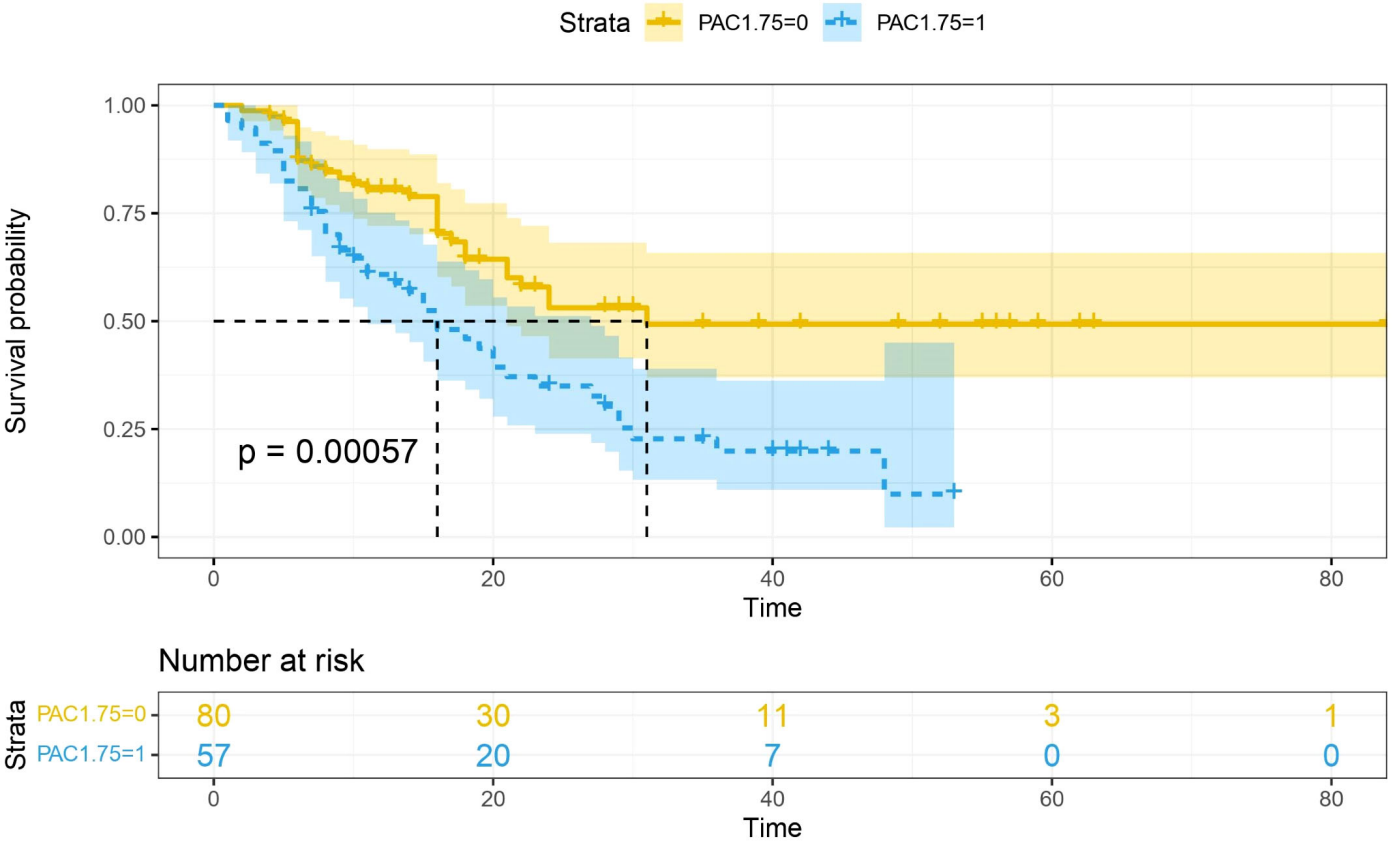
Survival curves stratified by lactate concentration. The blue curve represents the survival probability of patients with LAC > 1.75, and the yellow curve represents the survival probability of patients with LAC < 1.75. It was found that the survival probability of patients with LAC > 1.75 mmol/L was significantly reduced (p =0.00057).

**Figure 3:**
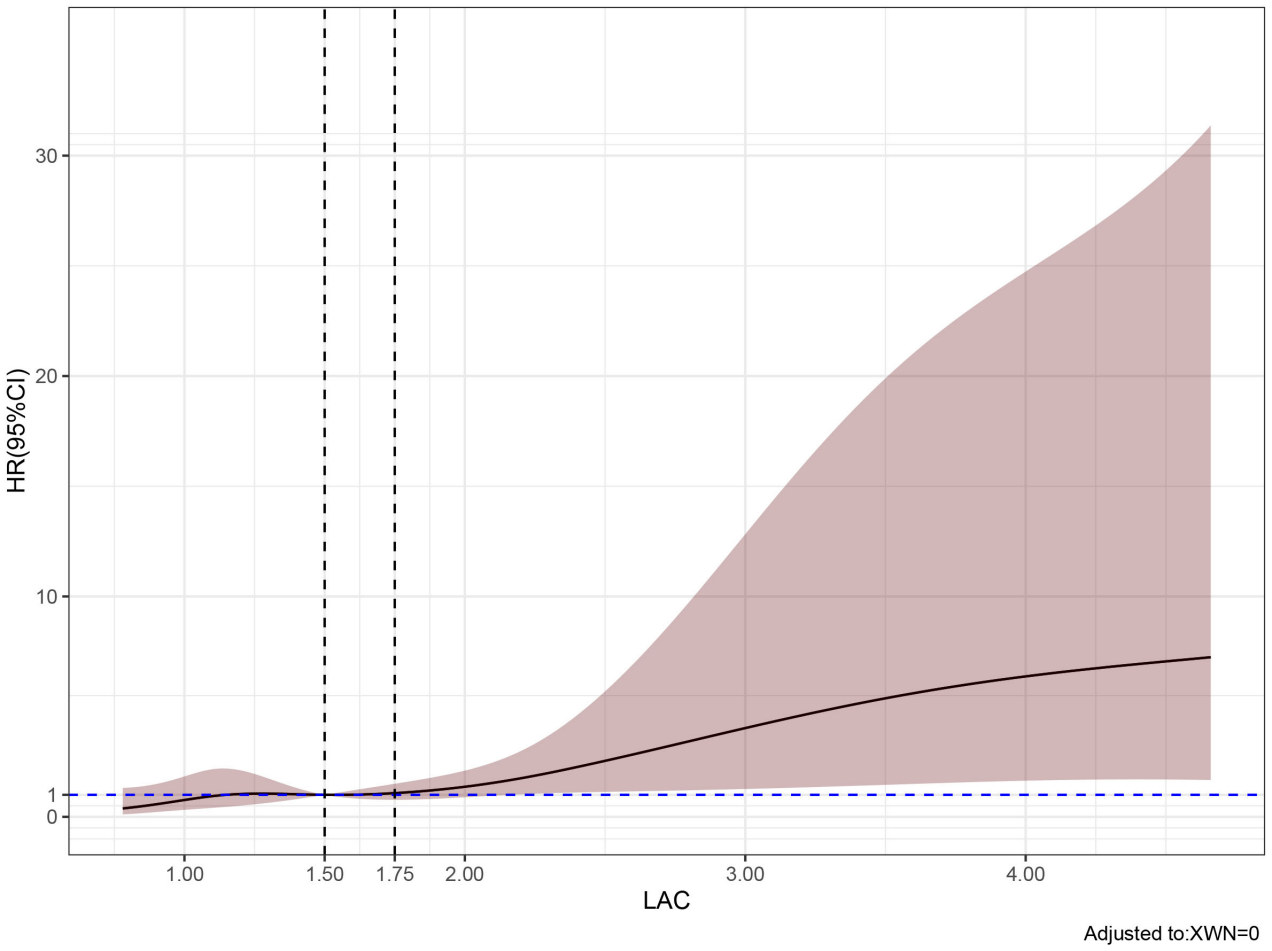
Predicted the correlation between LAC correlation and all-cause mortality in ICU patients with COVID-19. The risk ratio was expressed as a solid line, and 95% CI was shown as a shaded area. When the LAC concentration reaches and exceeds 1.5-1.75 mmol/L, the mortality rate of patients begins to increase rapidly.

Multivariate analysis showed that, excluding the interference of other factors, LAC level was an independent outcome predictor for ICU patients with COVID-19 (odds ratio [OR], 0.05; 95% confidence interval [CI], 0–0.1; *P* = 0.047). For LAC level, the AUC was 0.70, and the cut-off value was 1.75 mmol/L. The sensitivity and specificity were 57.7% and 75.8%, respectively. Using a cut-off value of 1.75 mmol/L (**Figure 1**) to classify patients as having high or low LAC levels, it was found that the survival probability of patients with a LAC level >1.75 mmol/L was significantly decreased (*P* = 0.00057) (**Figure 2**). When the patient’s LAC was > 1.75 mmol/L, their risk of death increased significantly (crude OR, 1.41; 95% CI, 1.2–1.65; *P* < 0.001), even after adjusting for age and gender (adjusted OR, 1.36; 95% CI, 1.15–1.58; *P* < 0.001) (**Table 3**). Additionally, we used the RCS function to flexibly model and visually predict the relationship between LAC level and all-cause mortality in patients hospitalized in the ICU with COVID-19, starting with a relatively stable risk of all-cause mortality until LAC concentrations reached 1.5–1.75 mmol/L, at which point all-cause mortality began to increase rapidly (non-linear *P* < 0.001) (**Figure 3**).

**Table 3:** Associations between LAC>1.75mmol/L and outcomes.

	Crude			Adjusted^**^		
	OR	95% confidence interval	P	OR	95% confidence interval	*P*
SOFA-D7	8.1	0.89-73.1	0.06	7.81	0.83-73.13	0.07
APACHII-D7	1.18	0.07-19.9	0.9	0.99	0.05-17.08	0.99
Time	0.02	0.0001-4.47	0.16	0.02	0.0001-5.39	0.18
Outcome	1.41	1.2-1.65	0.00004^*^	1.36	1.15-1.58	0.0003^*^

*p-value < 0.05. **Adjusted for sex, age.

### 3.3 Predictive role of LAC combined with lymphocyte change rate on prognosis

**Figure 4:**
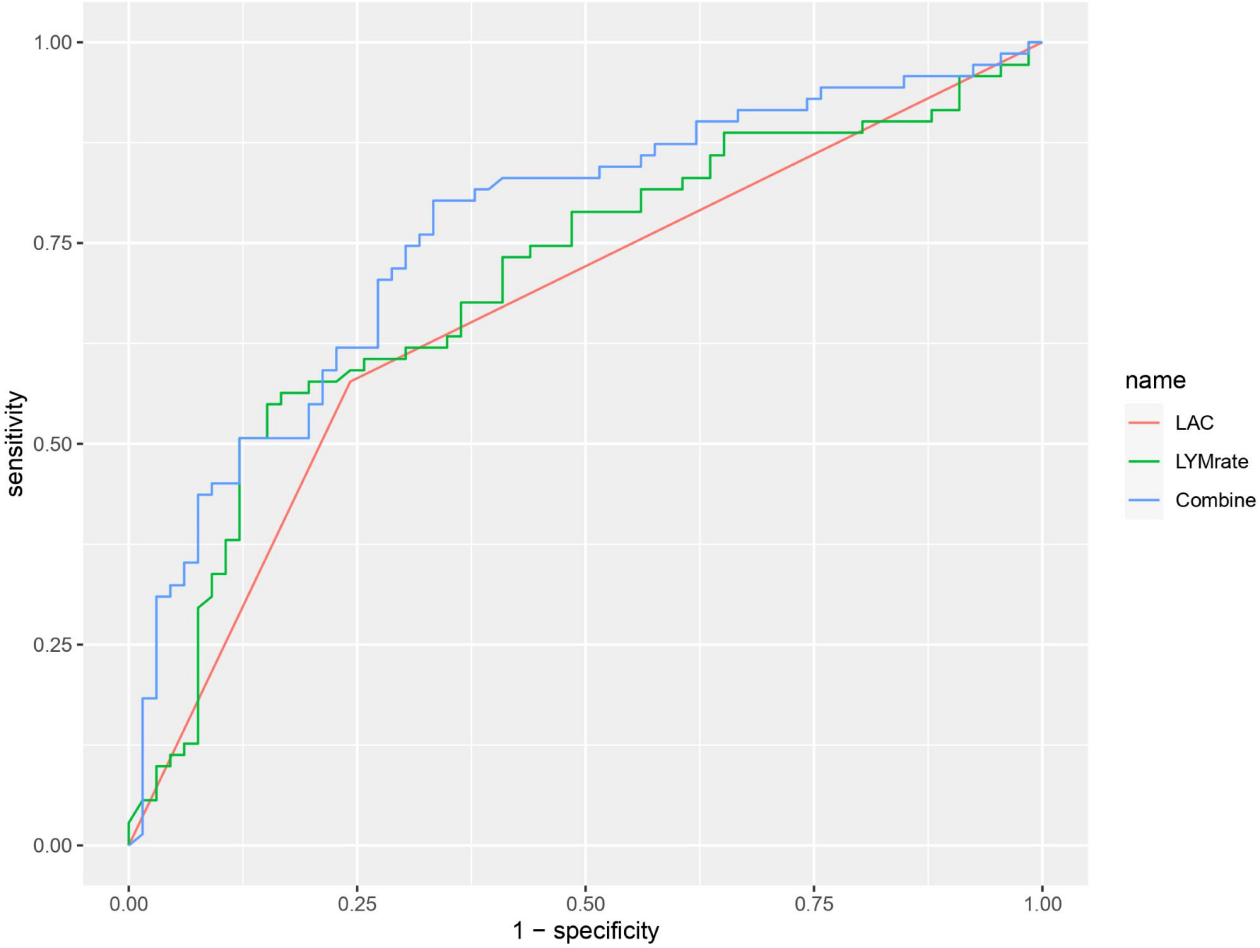
ROC curve graph showing the performance of three models: LAC (red), LYMrate (green), and Combine (blue). The x-axis represents 1-specificity and the y-axis represents sensitivity. The Combine model has the highest sensitivity across various thresholds.

The area under the ROC curve of the combination of lactate and lymphocyte change rate (AUC 0.7629) was significantly higher than that of lactate (AUC 0.70) and lymphocyte change rate (AUC 0.648) alone for prognosis (P < 0.05) (**Figure 4**).

### 3.4 Mediation analysis

**Figure 5:**
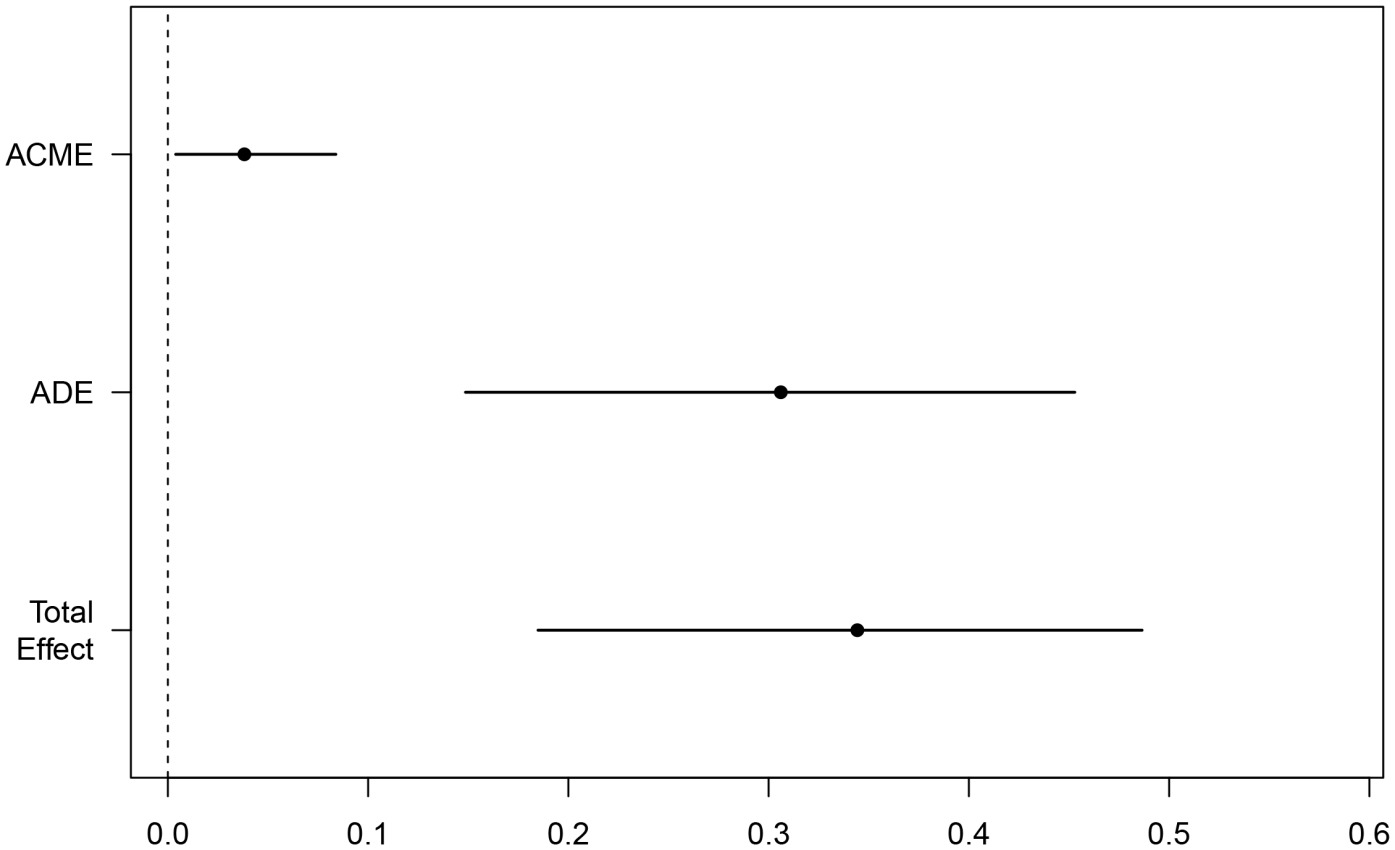
Mediating effect of NLR-mediated mediation of LAC on mortality in ICU COVID-19 patients; Total Effect, LAC combined with NLR mediated effect; ADE, LAC mediated effect; ACME, NLR mediated effect.

**Figure 6:**
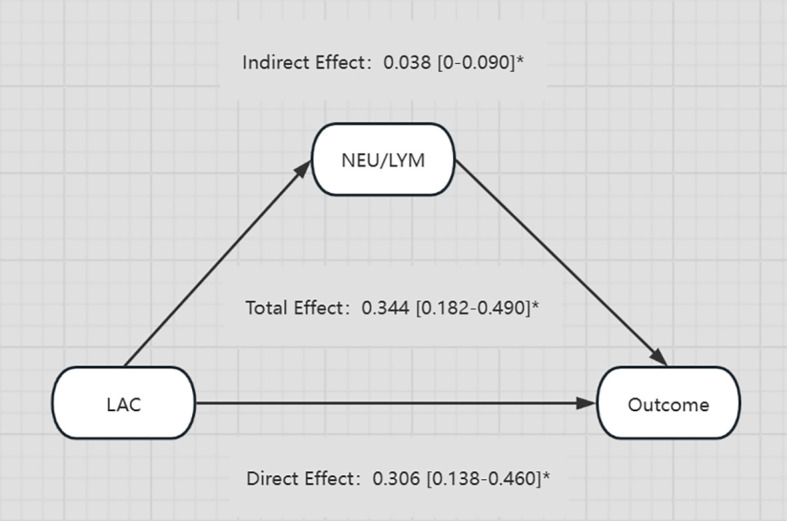
Diagram illustrating the relationships between LAC, NEU/LYM, and Outcome. LAC has a direct effect of 0.306 and an indirect effect through NEU/LYM of 0.038 on the Outcome. The total effect from LAC to Outcome is 0.344. All effects are provided with their respective confidence intervals and marked with an asterisk. *p-value < 0.05.

Considering the complexity of the reasons behind an increase of LAC in the body, and the corresponding relationship of LAC with the body’s immune and inflammatory responses, we evaluated in the present study whether or not there is a relationship between elevated LAC levels and other indicators, and whether that relationship has an enhancing or weakening effect on the relationship between LAC level and outcome. CMA is a method used to distinguish the total effect of something as the direct and indirect effect, where the indirect effect on an outcome is mediated by intermediate factors. CMA produces an average causal mediation effect (ACME), average direct effect (ADE), and total effect (Chen et al., 2020). Prior to the intermediate mediation analysis, the association between pre-specified mediators (including routine blood tests, biochemistry, coagulation, blood gas, and other related indicators) and prognosis was evaluated. Among the 15 clinical test indicators related to prognosis, only NLR showed a robust mediating effect after complete adjustment. The direct effect of LAC level and adverse outcomes (hazards ratio [HR], 0.306; 95% CI, 0.138–0.460; *P* < 0.001) was strengthened by the indirect effect of NLR (HR, 0.038; 95% CI, 0–0.090; *P* < 0.05), resulting in a greater total effect (HR, 0.344; 95% CI, 0.182–0.490; *P* < 0.001) (**Figures 5**, **6**).

### 3.5 Correlation analysis between P/F and lactate and lymphocyte change rate

**Figure 7:**
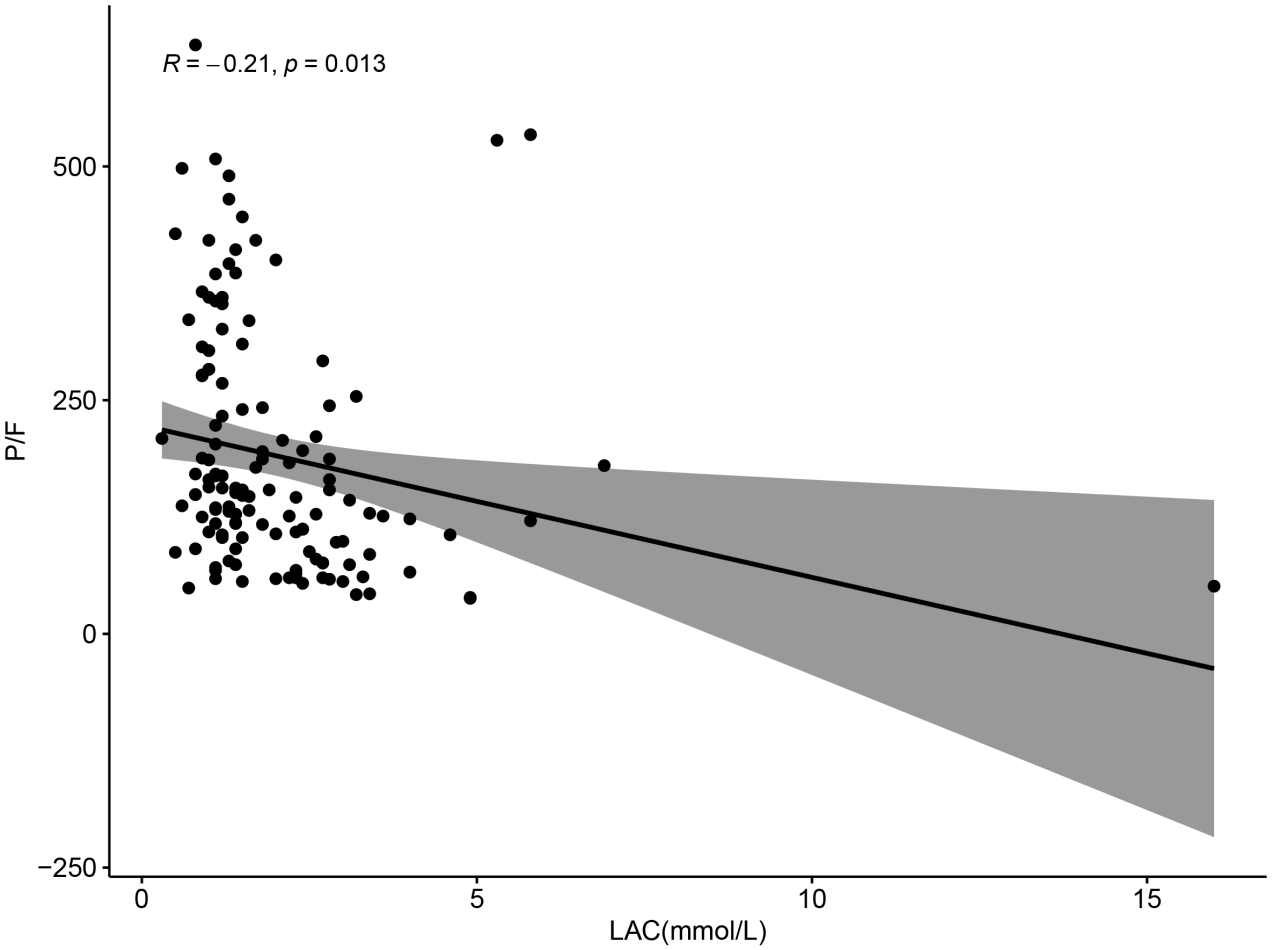
Scatter plot showing the relationship between LAC (mmol/L) and P/F ratio, with a negative correlation (R = -0.21, p = 0.013). Data points are scattered, and a trend line with a shaded confidence interval indicates a downward trend.

**Figure 8:**
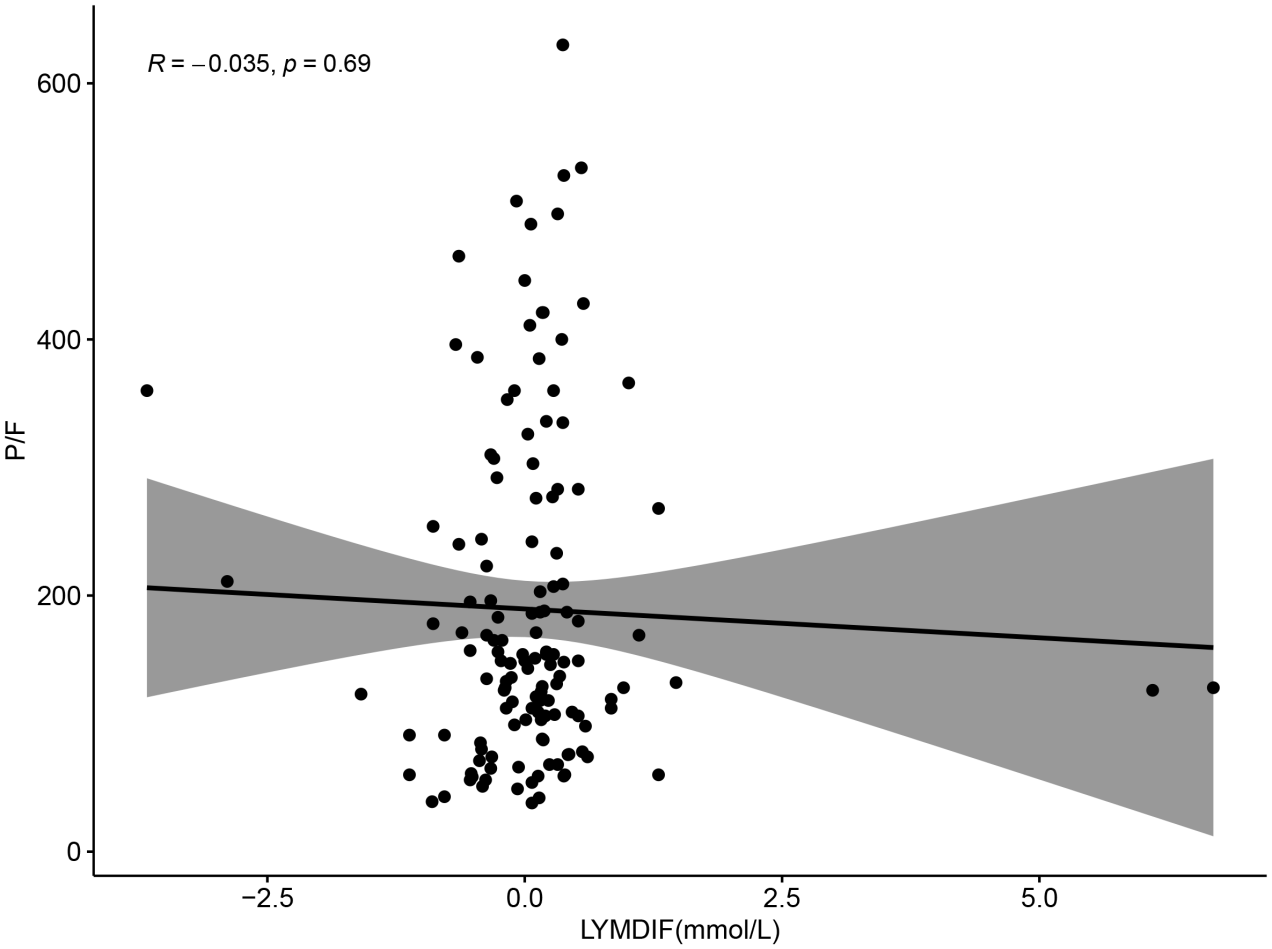
Scatter plot showing the relationship between LYMDIF (mmol/L) and P/F ratio. Data points are scattered with a best-fit line and shaded confidence interval. The correlation coefficient R is negative 0.035 with a p-value of 0.69, indicating no significant correlation.

Correlation analysis of P/F with lactate and lymphocyte change rate revealed a negative correlation between P/F and lactate (R=-0.21, P = 0.013), which was statistically significant (**Figure 7**). And although there was a negative correlation between P/F and lymphocyte change rate, it was not statistically significant (R=-0.035, P = 0.69) (**Figure 8**).

## 4 Discussion

In a retrospective analysis of patients with COVID-19 admitted to our comprehensive, respiratory, and infection ICUs during the COVID-19 pandemic, the predictive value of LAC level combined with the weekly LYM change rate for determining prognoses was greater than that of either alone, which is of practical value for judging the prognosis of ICU patients with COVID-19. The risk of death in patients with a LAC level >1.75 mmol/L on the day of admission to the ICU was significantly higher than that in patients with lower LAC levels. The increase in LAC, however, was not found to be associated with respiratory-related indicators, which may, in turn, be related to microcirculation disorders due to SARS-CoV-2 infection.

Arterial blood LAC level is used as a routine parameter of clinical evaluation in ICU patients, as increased LAC levels are common in critically ill patients, and are often used to assess disease severity, monitor and guide treatment, and determine prognosis. For example, The Sepsis-3 guidelines include high LAC levels (> 2 mmol/L) as a diagnostic criterion for septic shock, and recommend normalizing LAC levels as a goal during early fluid resuscitation (Rhodes et al., 2017). Traditionally, it has been believed that an increase in blood LAC is the result of anaerobic metabolism due to tissue hypoxia; however, increased LAC concentrations can also be found in non-hypoxic states of the body, such as a decrease in clearance due to kidney or liver dysfunction (Kumar et al., 2025), an increase in aerobic glycolysis (Chen et al., 2025), and beta-adrenergic stimulation (Rimachi et al., 2012), as a result of the interaction of oxygen supply, tissue metabolism, and blood clearance. In the early stages of the COVID-19 epidemic in Wuhan, it was found that about 84% of elderly patients (≥ 60 years old) had elevated LAC levels (Li P. et al., 2020). A data analysis of 2,860 elderly patients admitted to the ICU with COVID-19 showed that the initial LAC levels of those that did not survive were significantly higher than in those that did survive, increased serum lactate on the first day was related to a significant increase in ICU mortality, as well as 30-day and 3-month mortality, and ICU mortality was negatively correlated with a decrease in the LAC level (Bruno et al., 2021). In a Greek single-center study, Alice et al (Vassiliou et al., 2020) proposed that a cut-off value of 1.85 mmol/L for LAC level could predict mortality in ICU patients with COVID-19. The statistical analysis performed in the present retrospective study found that 1.75 mmol/L could be used as a critical value to judge the prognosis of ICU patients with severe COVID-19, and found that lactate >1.75 mmol/L was significantly correlated with the patients’ prognoses (*P* < 0.001). The difference in this cut-off value may be due to regional differences, different prevalent strains, or other factors.

Researchers have studied the pathogenic mechanism of SARS-CoV-2, the pathogen that causes COVID-19, and have found that the virus mainly exerts its toxic effect by binding to the host receptor membrane ACE2 through its spike S-glycoprotein (Gheblawi et al., 2020), while ACE2 is expressed in a variety of cells in the respiratory tract, so that the SARS-CoV-2 directly infects the bronchial and alveolar epithelial cells, causing lung tissue damage and affecting the ventilation and air exchange function of the body (Sungnak et al., 2020). In the present study, however, we found that partial pressure of oxygen (PO_2_) and Oxygen saturation(SO_2_)were lower, while partial pressure of carbon dioxide (PCO_2_) and FIO_2_ were higher, in patients who died of severe COVID-19 than in the surviving patients, although none of the differences were significant. Additionally, we observed no significant relationship between these values and prognosis, even after excluding interference from other factors through multifactorial regression analysis (*P* > 0.05), while LAC was found to be significantly associated with prognosis (*P* = 0.047). In regards to the source and clearance of LAC, an increase in LAC throughout the body may be caused by insufficient tissue perfusion. (Li H. et al., 2020) found that approximately 71.4% of patients who died of COVID-19 had disseminated intravascular coagulation, evidenced by the observation of a thrombus in the microvascular system upon autopsy (Wichmann et al., 2020). Tang et al (2020a; 2020b). reviewed coagulation parameters in COVID-19 patients, and proposed that FDP and DD levels were significantly higher in deceased than surviving patients, and that patients with DD levels exceeding six times the upper limit of normal had an increased mortality rate, suggesting that although other factors may contribute to the elevation of LAC in the body, reduced tissue perfusion due to thrombosis caused by microcirculatory thrombi may be the primary cause of increased LAC levels in patients with COVID-19.

Since ACE2 is highly expressed in arteriovenous endothelia, viruses cause damage and apoptosis to the vascular endothelia by binding to ACE2, triggering endothelial inflammation, and subsequently, thrombus formation (Varga et al., 2020). Simultaneously, ACE2, as a key link in the renin-angiotensin-aldosterone system (RASS), plays a role in vasodilation and anti-inflammatory actions; therefore, when ACE2 is blocked by the virus and its expression is downregulated, the microcirculatory blood flow decreases, resulting in inadequate circulatory perfusion of the tissue (Patel et al., 2016; Hanff et al., 2020), as oxygen exchange occurs in the microcirculatory system, and oxygen consumption is limited when systemic oxygen delivery is reduced (Wang et al., 2023). Moreover, SARS-CoV-2 infection can cause mitochondrial reactive oxygen species (ROS) production and dysfunction, inducing hypoxia-inducible factor-1 (HIF-1) activation, reduced oxidative phosphorylation, and increased aerobic glycolysis, resulting in an increase in LAC levels (Kumar et al., 2021; Tian et al., 2021; Yang et al., 2022). Additionally, a leftward shift in the oxygen dissociation curve can be observed in COVID-19 patients (Böning et al., 2021), indicating an increased affinity between hemoglobin and oxygen. Even if the oxygen content in a patient’s blood is guaranteed, the ability of the tissue to utilize oxygen decreases, which might explain the lack of significant difference in PO_2_ between the deceased and surviving patients in the results of the present study, although there was a significant difference in LAC levels. In another retrospective study of COVID-19 patients, (Tang et al. 2020b) found that mortality in patients with COVID-19-associated coagulopathy who were treated with heparin was significantly lower than in those who did not receive anticoagulation. Li et al (Ma et al., 2022) demonstrated, for the first time, that low molecular weight heparin (LMWH) can reduce plasma LAC levels in patients with COVID-19, which may be associated with a reduction of microthrombus formation. Therefore, the authors of the present study suggest that even in patients with COVID-19 whose initial and most severely affected organ is the lungs, the elevation of LAC may not be entirely to ventilation and air exchange dysfunction caused by pulmonary thrombi, but may also be due to inflammation and immune response-induced alterations in the coagulation system of the body. These changes affect the microcirculatory blood flow, leading to insufficient tissue blood supply and reduced oxygen utilization, subsequently resulting in increased LAC production due to increased aerobic glycolysis.

The body’s immune system is composed of an innate immune response, which is induced by neutrophils and is the body’s first line of defense against invading pathogens, and an adaptive immune system, which is dominated by lymphocytes, including B-cells as well as T-cells, which provide specific antigenic responses to the body (Song et al., 2021; Buonacera et al., 2022). The two coordinate with each other and reflect the balance of the body ‘s immunity. Under normal conditions, T cells activated in response to inflammation, such as regulatory T cells (Treg), inhibit the production of the pro-inflammatory cytokine IL-6 by neutrophils, and transform into a large number of anti-inflammatory factors, such as IL-10, TGF-β, and indoleamine 2,3-dioxygenase (IDO)), which induce apoptosis in neutrophils and counteract the excessive inflammatory process mediated by neutrophils (Lewkowicz et al., 2013; Schönrich et al., 2020). In the early stage of SARS-CoV-2 infection, neutrophils are stimulated to activate and secrete large amounts of reactive oxygen species (ROS) (Jiang et al., 2025; Schönrich et al., 2020), which induces oxidative stress and increases the formation of neutrophil extracellular traps (NET), thereby suppressing the activity of T lymphocytes (Tripathi et al., 2018). Moreover, SARS-CoV-2 can directly affect lymphoid organs and cells through ACE2, causing the destruction and apoptosis of lymphocyte (Gu et al., 2005). The virus can also cause an innate immune system overreaction and cytokine storm in the body, resulting in a substantial decrease in peripheral lymphocyte counts (Colantuoni et al., 2020). Similar to the results of previous studies, the results of the present study found that the majority of ICU patients diagnosed with COVID-19 had lymphopenia, and that there were significant differences in LYM counts between the deceased and surviving patients. To better determine the relationship between LYM counts and disease prognosis, we collected LYM counts on the first and seventh days of the ICU admission, and calculated the rate of over the course of one week (change in LYM count throughout the week/LYM count on day of admission) to define the immune dysregulation caused by COVID-19. Statistical analysis revealed that the LYM change rates were significantly correlated with patients’ prognoses (P < 0.001), and that the predictive value of this index combined with LAC for prognosis was greater than that of either index alone. Similar studies have been conducted on the impact of LAC and LYM counts on the prognosis of patients with COVID-19. For example, Wang et al. (2021) have pointed out that LYM counts were nonlinearly associated with the risk of death in patients hospitalized with COVID-19. With the decrease of LYM counts (< 0.95 × 109/L), the risk of death in hospitalized patients with COVID-19 will gradually increase. Vassiliou et al. (2020) found that initial blood lactate in COVID-19 patients admitted to the ICU served as an independent outcome predictor and that the time course of LAC reflected changes in organ dysfunction during hospitalization, which correlated with adverse clinical outcomes. Unlike previous studies, considering the continuous changes of lymphocytes in patients with severe COVID-19, the change rate of lymphocytes within one week was calculated, and the patient data collected in this study were from the pandemic period after the implementation of the COVID-19 open management policy in China. At this time, the popular strain was mainly Omicron, which had higher infection rate and immune evasion than other strains (Parsons and Acharya, 2023).

LAC is considered a functional chemical that can affect the body’s immune response, which may also explain why NLR mediated the of LAC on the mortality of severe patients in the causal mediation analysis of this study; The study found that in severe COVID-19 patients, NK cells were strongly activated but their function was significantly reduced, mainly manifested as dim ­CD56dim phenotype cells, compared to bright phenotype ­CD56bright cells, and the former has a significant-inflammatory and cytotoxic effects (Maucourant et al., 2020; Osuchowski et al., 2021). While under low lactate levels, such as in the recovery period after exercise, the ratio of CD56bright to CD56dim cells increased significantly (Timmons and Cieslak, 2008; Gupta, 2022). In cancer patients, it was found that the acidosis induced by elevated LAC can significantly inhibit NK cell activity and reduce the level of interferon-γby it, while correcting acidosis can promote the degeneration of tumor cells (Greppi et al., 2024); T cells have specific transport proteins to sense lactate, inhibit its mobility, and increase the conversion of CD4+ T cells to an IL-17+ fraction, while reducing the cytolytic activity of CD8+ T cells (Pucino et al., 2019). In sepsis patients, increasing glycolysis, promoting ATP production, and relieving lactic acidosis can significantly offset the immunosuppressive effects of LAC and enhance the immune function of the body (Liu et al., 2024), so may be possible to explore the treatment of severe COVID-19 patients through corresponding LAC blocking strategies, so as to improve their prognosis.

Multivariate analysis revealed that the SOFA score also had a predictive value for the prognosis of patients with COVID-19; however, considering that the present study was retrospective in nature, the SOFA score included a subjective evaluation of the state of consciousness, and the SOFA score, as well as LAC, both indicated organ dysfunction in the body. Therefore, the present study did not include an evaluation of lactic acid combined with the SOFA score on the prognosis of patients with COVID-19; however, we did evaluate the predictive value of the combination of clinical detection indicators. In the present study, an increased NLR, when associated with increased LAC levels, mediated an increased risk of poor prognosis in ICU patients with COVID-19, which may be attributed to the body’s systemic inflammatory and excessive immune response, with an increase in NEUs and a decrease in LYMs, while LAC regulates the immune and inflammatory responses of the body.

## 5 Conclusions

The present study analyzed clinical indicators of patients admitted to the ICU during the Omicron variant phase of the COVID-19 pandemic, and found that clinical tests, such as LAC level, LYM change rate, and NLR, had predictive value for the prognosis of ICU patients with COVID-19. The cut-off value of LAC was found to be 1.75 mmol/L, and the prognosis of patients with a LAC level >1.75 mmol/L was significantly worse than that of patients with a LAC level <1.75 mmol/L. LAC level, combined with the weekly LYM change rate, had the greatest predictive value for the prognosis of patients in the ICU diagnosed with COVID-19. We suggest, therefore, that the elevated LAC levels may be caused by insufficient tissue blood supply due to microcirculatory thrombosis, rather than impaired pulmonary ventilation.

## 6 Limitations

The present study had a few limitations. First, the patient data collected in the present study came from different ICUs within the same hospital and all the patients included were from China; therefore, selection bias and variability in detection may be present. Secondly, the present study was retrospective in nature, encompassing a smaller sample size, which may limit the interpretation of the results. Moreover, because the subjects were ICU patients who may have been treated in the general ward prior to being admitted to the ICU, not all patients were admitted directly from the emergency department, which may have affected the results of the present study. Finally, the patients’ viral loads were not measured or statistically analyzed. Additionally, whether or not patients received the vaccine before the onset of the disease was not included in the present study. These factors, therefore, may have impacted our results.

## Data Availability

The datasets presented in this study can be found in online repositories. The names of the repository/repositories and accession number(s) can be found in the article/Supplementary Material.
